# Ping pong for health: the meaning of space in a sport based health intervention at the workplace

**DOI:** 10.1080/17482631.2019.1689602

**Published:** 2020-10-25

**Authors:** Krister Hertting, Mats Holmquist, James Parker

**Affiliations:** Department of Health and Welfare, Halmstad University, Halmstad, Sweden

**Keywords:** Health promotion, sport-based intervention, well-being, workplace, phenomenology, lived space, physical activity, learning, social relations, health culture

## Abstract

**Purpose**: This is a study on a sport-based intervention, with a focus on physical activity, social relations, and learning, to promote health and well-being in the workplace lived space. Lived space is situated and associated with social and cultural conventions which affect the quality of the perceived space at work. The aim of the paper is to elucidate the participant’s experiences of the intervention and how health and well-being were affected.

**Methods**: The intervention was conducted with employees from the warehouse of a company within the retail sector. The design consisted of one initial workshop as a baseline, a sport-based intervention, three group interviews, and a final workshop. A hermeneutic phenomenological analysis focused on experiences of the intervention and the meaning of the workplace as the lived space.

**Results**: Three themes emerged in the analysis; *Expressing positive individual effects, Expressing improved work environment* and *The meaning of the workplace as lived space*. The themes are discussed in relation to three basic health foci: physical activity, social relations and learning.

**Conclusion**: The workplace as a lived space offers a valuable opportunity for sport-based interventions that improve health and well-being through physical activity, social relations, and learning.

## Introduction

Poor employee health such as physical inactivity and being unable to manage stress are associated with an increased cost for the employer (Rajaratnam, Sears, Shi, Coberley, & Pope, 2014). A number of risks associated with increased costs are considered to be modifiable, that is with the correct intervention strategy employee health can be improved and employer’s costs can be reduced. Adopting a “healthy organizational” culture through health programmes, with strong senior and middle management support, and using interventions can change these modifiable risk factors (Rajaratnam, Sears, Shi, Coberley, & Pope, 2014). The workplace is a space that offers considerable opportunity for health promotion initiatives, such as those which promote sustainable physical active lifestyles, which target the adult population (Johnson et al., 2018). Some key differences exist between public health and workplace health, for example, workplace initiatives can be tailored to meet specific industry segments and demographic groups in a way that public health initiatives cannot (Karanika‐Murray & Weyman, 2013). However, companies can express a disinclination to “meddle” in their employees lives and do not always consider it appropriate to make suggestions relating to employees’ lifestyle changes (Pescud et al., 2015). Nevertheless, work places have the potential to make a three-to-one return on investment by reducing medical and absenteeism costs, period and improve employee work efficiency (Rajaratnam et al., 2014).

The organizational culture can comprise/constitute a strong or weak culture of health (Taylor, Suminski, Das, Paxton, & Craig, 2018), which in turn affects the experience of the lived space (van Manen, 1997). Meacham Webb and Krick (2017) defined over twenty elements of a healthy culture that influence employee health and wellbeing. Among those are employee involvement and empowerment, training and learning, relationship development and a sense of community. Payne, Cluff, Lang, Matson-Koffman, and Morgan-Lopez (2018) identified that relational elements of employee engagement, co-worker support and leadership support tend to be associated with a culture of health. Rahrig Jenkins, Fakhoury, Marzec, and Harlow-Rosentraub (2014) found that two main perceptions of a culture of health were leaders/supervisors showing interest in health and well-being, and colleagues participating in healthy behaviours and setting good examples. Understanding the root causes of success, individual and organizational, is considered to be essential in order to amplify those things that will help build for example a better workplace (Ghaye et al., 2008). One method that focuses on stimulating these root causes and that has been shown to be successful in workplace health promotion is through the use of empowering settings and participatory approaches (Torp, Eklund, & Thorpenberg, 2011). In this project we view empowerment, in accordance with Wåhlin (2017), as bringing individual and social aspects together leading to *“increased sense of coherence and control over the situation, personal and/or professional development and growth, and increased comfort and inner satisfaction”* (p. 172).

One means to stimulate empowering setting is through learning interventions. Learning interventions can be categorized according to their primary purpose of learning as interventions aiming at developing personal resources, professional capabilities, leadership skills and improve organizational effectiveness Watson, Tregaskis, Gedikli, Vaughn, and Semkina (2018). In general, learning had a positive impact on well-being, which is also confirmed by Perkins and Williamon (2014). The intervention in present study mainly focused on personal resources, which could be compared to Dreyfus and Dreyfus (1999), Dreyfus (2004)) model of learning based on Merleau-Ponty’s (2002/1962) intentional bow and the embodiment of knowledge. By focusing on continually learning, motivation is a characteristic of the embodiment of knowledge (Dreyfus, 2004; Dreyfus & Dreyfus, 1999). Sports participation can improve physical and mental health (Levermore, 2008), and sports offer a unique opportunity for physical activity interventions to be combined with learning intervention. There are encouraging findings (Levermore, 2008), particularly among children, that participation sport can improve wellbeing and social relations there is a lack of paucity of empirical evidence describing the impact of sports-based physical activity interventions in the workplace.

## Rationale and aim

Physical activity (Delisle, Werch, Wong, Bian, & Weiler, 2010; Warburton & Bredin, 2016), social relations (Lamu & Olsen, 2016) and learning (Perkins & Williamon, 2014; Watson et al., 2018) have positive impacts on physical and psychological well-being. Is it possible to get these positive impacts through a sports-based table-tennis intervention at the workplace? How does the meaning of the workplace as a lived space affect the impact of the intervention? The aim of this paper is to elucidate the participant’s experiences of a sports-based physical activity intervention at the workplace and how health, well-being, social relations, and organizational culture were affected in relation to lived space.

## Theoretical framework

Theoretically, this paper is informed by the phenomenology of the life-world, focusing on lived space. Lived space is not equivalent to physical space; it is an unreflected and non-verbal space (Bollnow, 1994; Langeveld, 1983; van Manen, 1997). Lived space is situated and associated with social and cultural conventions which affect the quality of the perceived space (for example at work, at home, by the table-tennis table). People influence and are influenced by the mood of the space. According to Bengtsson (1998), all spaces have a certain penetrating mood that depends, for example, on what tasks are to be performed, which other people are in the room, time of day, and mindset. Most importantly, the room should not be reduced to either an inner space or just a physical space. The lived space is in the meeting between these; the subject inhabits the room and constructs the meaning in the space, or in other words the lived body inhabits the space (Merleau-Ponty, 2002/1962).

Shilling (2003) considers the body as the source of understanding society. Through our physicality, we are included in and excluded from communities. The space changes and evolves over time, which according to Eichberg (1998, 2016) means that the body is a subject of social change and the body and physical activity are objects for different ideas and ideologies. In modern society, time, standardization, measurability, competition and results are important factors, and competitive sports evolved in the age of modernity (Eichberg, 1998, 2016; Gustavsson, 1994). Central components of competition are time and performance as absolute, measurable, quantifiable and one-dimensional. This means that space according to Eichberg (1998) becomes monofunctional, that is, it is well defined for current activities, and the body is shaped by training for high performance. This means that spaces for sports are not natural environments, but created specifically for the current time and location factors:

Sport is thus a visible, living metaphor of modernity: making the world measurable, making the human being productive and ‘developing’ space under the dominance of ‘dynamic’ time. The one and dominating rationality finds its expression in pure form through the time-space patterns of sport. (Eichberg, 1998, s. 153)

When designing a sport-based intervention it is important to understand that history and ideologies can affect the space and bodily experience of the participants. Table-tennis is situated in a certain social space and connected to a variety of values, such as enjoyment and play but also performance and competition, which can be challenging in a health promotion intervention

## Research design

An intervention based on physical activity, social relations, and learning was developed with the aim to improve health and well-being by introducing table-tennis in the workplace. The intervention was conducted in the warehouse of a company within the retail sector where their own sports facility- and table tennis room at the workplace. The reason for choosing table tennis as the activity was that the game can be played at all levels, from novice to expert, it can be facilitated at the workplace since it does not require much physical space, it is a physical activity played together in a social setting, and everyone can learn and improve regardless of their background. Since previous studies emphasize management support as crucial (Payne et al., 2018; Rajaratnam et al., 2014) the first step was to reassure their support. The workplace management was then responsible for recruiting participants and an open invitation was shared to all employees at the company, from managers to the warehouse workers. The workplace had approximately 150 employees, and thirteen agreed to participate. The participants came from different groups of employees and had various backgrounds in table-tennis and sport in general with different motives to participate in the intervention.

The intervention was scheduled over twelve weeks and consisted of an initial workshop, five table-tennis sessions once a week for five weeks led by two coaches, a follow up and a final workshop summarizing and validating results and pointing out future directions for health at the workplace (see Table 1).

After the table-tennis sessions and before the Workshop 2 all participants were invited to group interviews. All participants accepted the invitation, nine participated and four did not have the possibility to leave their work tasks at the time form interview. The participants had shared experiences and were acquainted through the intervention, so it was decided to conduct focus group interviews, which is an arranged group discussion, where the interaction between the participants means that the dynamics of the conversation is greater than in the case of a formal interview with an informant (Vaughn, Schumm, & Sinagub, 1996; Wibeck, 2010). Semi-structured focus group interviews inspired by Kvale (2007) were conducted. An interview guide with four themes was constructed: the table-tennis intervention, prerequisites for participation, consequences of participation (for the individual and the group), and lessons learned for the future. The interviews were conducted in a conference room at the workplace, which was a familiar environment and created a relaxed atmosphere (Kvale, 2007). Two of the researchers participated in every interview, one was responsible for the interview and the other took notes and filled in with questions when needed. The interviews, which lasted between 45 and 60 minutes, were recorded and transcribed verbatim. The interview data is the base of analysis in the paper. One demarcation of the study was to study health promotive effects of a sport-based intervention, which means that the study did not actively intervene with questions concerning for instance job design/role, workload, systems of reward, leadership style and the underpinning climate. However, when employees raised these questions during interviews this was elucidated.

## Analytical framework

The inductive data analysis was inspired by van Manen’s (1997) hermeneutic phenomenological method, supported by the framework of phenomenological analysis developed by Lindseth and Norberg (2004). The phenomenon, in this case, was employee’s experiences of a sport-based intervention, and the process of analysis was guided by an understanding of this phenomenon. The starting point of the analysis sought to “elucidate employee’s experiences of a sport-based intervention and how health, well-being, and organizational culture were affected”. Through other people’s sharing of their experiences and reflections, we can have opportunities to deepen our understanding of a certain phenomenon or a certain aspect of human life. This means closeness to the phenomenon as well as to the context studied. Using the participants’ stories is giving the employees an opportunity to define their experiences by themselves (Johansson, Almerud Österberg, Leksell, & Berglund, 2016), allowing them to describe their own perspectives. It also means an interrelation between the collection and analysis of data, which often, according to van Manen (1997), turns out to be a parallel process. Nonetheless, during the analysis, consideration of difficulties in interpreting the material was highlighted. The employee’s responses might, for instance, have been affected by the power relations created by managers and employees or us as researchers (Kvale, 2007).

The process of analysis began with all researchers conducting *naïve reading* (Lindseth & Norberg, 2004) before the first discussion of the results. During discussions, reflections were made and similarities and differences were noted and discussed, and the structural analysis started, with the analytical steps to *identify meaning units, sub-themes* and *themes* (Lindseth & Norberg, 2004). All interviews were read by asking “what does this say about the employee’s experiences of a sport-based intervention and how were health, well-being, and organizational culture affected?”. Patterns were identified and constituted the meaning units. The meaning units were then compared and discussed. This selective and detailed discussion, with reflections on these meaning units, their differences and similarities, resulted in a first understanding of what it is that constitutes the nature of employee’s experiences, the sub themes. The analysis continued with a focus on individual sections, as well as the whole of the interviews, in order to better understand patterns of meaning, which led to three main themes (see Table II). To enhance validity, quotations were identified and used when describing the results.

**Table I.** Intervention activities

Activity	Intervention week
Workshop 1: Situation profile; physiological and psychosocial, 13 participants	Week 1
Table tennis practice with two coaches, once a week, 45 minutes, two groups (six participants in each group)	Week 2–6
Monitoring profile; physiological and psychosocial	Week 8
Group interviews; three groups in total 9 participants	Week 9
Workshop 2: Feedback and validation of results, discussion of future goals and directions for sport-based health interventions at the workplace, in total six participants	Week 12

**Table II.** Overview of results

Themes	Sub-themes
Expressing positive individual effects	Feeling enjoyment in learning and competing in table-tennis Feeling increased social connectedness Improved general well-being
Expressing improved work environment	Acknowledging increased work cohesion Feeling increased work motivation Recognizing increased work ability
The meaning of the workplace as lived space	Having accessibility Being allowed to participate on working hours Having competition mood

## Results

Three themes, which will be presented and described below, emerged through the analysis; *Expressing positive individual effects, Expressing improved work environment* and *The meaning of the workplace as a lived space*. The participants in the current study can be grouped according to physically active (12 participants, women = 2 and men = 10) and sedentary (1 participant, man = 1). The physically active group can be further grouped as those motivated primarily by competition (10 men and 1 woman) and those motivated by learning (1 woman), where the latter participant chose to participate in the study because she wanted to learn a new sport (table tennis).

### Expressing positive individual effects

The employees who participated in this study found table tennis social, easy to get started, pretty exciting., and stimulated new non-work-related learning One of the most positive results for the individuals were the social and learning effects, which was especially emphasized by the already physically active group.

I learnt a lot about the mental side of the game. (P1).

Sport in the workplace space was experienced as giving an increased well-being through enhanced self-confidence and satisfaction of learning new things. Participants described for instance that learning that specific skill improved the game, and a general feeling of satisfaction.

One of the best things to see was that we learnt things and we learnt them quickly (P2)

The participants experienced that they were more positive after the training session and returned to their work tasks with more energy and the joy, which they experienced also spilled over on to, and affected, their colleagues.

I was probably a little happier afterwards. I felt more energized and not as lethargic as I usually do in the afternoon. It was a little kick of endorphins. (P3)

Table tennis was common ground and something to gather around and talk about at work. Colleges came to know each other more at the workplace, which facilitated contacts at work and participants experienced increased job satisfaction.

Table tennis has become something in common to gather around and talk about. You have come to know more people in the workplace, it has made contact easier at work. (P9)

The participants also experienced a stronger social context, they appreciated meeting colleagues from other departments in the company, which strengthened the social cohesion and enjoyment at the workplace (whilst at work).

We had another sense of community on the floor, we could chat and have a laugh. It was much a better atmosphere. (P4)

The physically active group was motivated by competition. They all had some previous experience of table tennis and enjoyed learning new skills, which helped them to become better players. Partly, to be able to cope with tricky situations within table tennis, and partly to have a greater chance of winning matches against colleagues and bosses. This was important for the group.

The physically active woman, who was not motivated by competition, enjoyed learning a new sport. She often ended up somewhat excluded from the competitive matches and thought the training sessions were often too hard (skill level) with too much focus on the participant with previous table tennis experience (physically active group motivated by competition).

I found it hard, I’d never played before, which wasn’t great. Things went a little too quickly. It would have been better if we’d been split up according to level (ability). (P5)

On the other hand, she shared the positive social benefits and thought that workplace happiness/wellbeing had improved.

You get to know people in a new way, in a way that isn’t connected to work, which is fun. (P5)

The sedentary “group” consisted of just one participant and, as such, the following analysis cannot be generalized but the results can nevertheless provide a ground/foundation for relevant reflections and information. The sedentary man was overweight and worked as a truck driver. He experienced positive results within the physical activity and social dimensions. Despite the relatively short intervention period consisting of only a few (five) training sessions, where it is unlikely the physical activity could lead to measurable changes in health and wellbeing the truck driver perceived and improvement in physical wellbeing alongside an improvement in social wellbeing.

Nah, I’m wasn’t that bothered about being active. But we’re going to start; one of our neighbors’ plays table tennis. He’s looking into the possibility of training at the arena…. Above-all for those of us who took part in the training (intervention). It will spread and then there will always be more that want to play. Hopefully, we will get even more people interested…. but it’s still in the early stages…. I would really like to use the grant the company gives us for healthcare on that, I could easily pay a bit myself if it was needed, to be able to get started. (P6)

In summary, participants expressed feeling enjoyment in learning and competing in table-tennis, feeling increased social connectedness and improved general well-being.

### Expressing improved work environment

The participants discussed the work organization and how they experienced an improvement in the work environment. One main benefit elucidated was social effects.

I barely ever spoke to Lena in the freezer department before. But now I usually try to say hello and ask her how she is. That means a lot. (P1)

Improved communication and cohesion between different departments within the company was also mentioned, and participants experienced that this could strengthen production and facilitate problem solving on a daily basis.

At the same time, you get to know them in a totally different way. You see loads of different sides to a person when you do these types of things. (P7)

The organizations logistics between loading and unloading and between freezer, fridge, and dry goods were experienced as creating social boundaries along with an “us and them” mindset which could cause difficulties and problems in normal daily routines. The intervention created new social spaces, which participants found helpful. In the long run, expanded social spaces and lower boundaries between groups of employees could merge, and this was acknowledged by the participants.

I thought it was good that there were people from the fridge, freezer, and high bay, I’ve come to know them and we’ve become closer: It’s easier to speak to someone from another department now that I know a few people from that department. (P5)

Sport in the workplace space improved the social and organizational work environment by strengthening cohesion and cooperation, well-being and the community. This was expressed in several ways, and the knowledge of the activity itself increased motivation to go to work.

Of course you’re happier if you have these types of activities, you actually want to go to work because it’s fun. (P1)

The support from managers was crucial, and one of the participating middle-managers explained:

In the beginning, the discussion was that “we should not do this” until it was turned to the advantage that it is, after all, to make our employees happy, give them positive energy and all this. That’s what we had to turn this to. Because it is still a cost for the company when it’s conducted during working hours. (P6)

The expanded social space contributed to positive organizational effects in the workplace environment, according to the participants.

Cohesion in the group, when we go out on the floor we have another community suddenly, we can talk and just chat like that, another mood on the floor. (P4)

By saying this, it could be suggested that an organization that establishes sport could use this in recruitment purposes, an added value to the ordinary work, trying to recruit new groups of employees.

What gets people to come is that they are interested and possibly think it’s important to do things together, you know be sociable (P5)

The participants experienced that it also served to prevent overuse injuries. The positive effects of physical activity for the participant with a sedentary lifestyle included a potentially improved physical work environment at the company.

Most of us who work here have to lift heavy things, so everybody should feel better if they did some sort of strength training or other training that can help us get stronger so we can then do what we need to do. (P5)

In summary, participants were acknowledging increased work cohesion, feeling increased work motivation and recognizing increased work ability. Based on participants experiences it could be suggested that if an organization can successfully implement sport at the workplace for employees with a sedentary lifestyle there is a possibility that days of absence caused by injury and illness could be reduced.

### The meaning of the workplace as lived space

The intervention took place at the workplace in a specific sport’s room. This space contributed to rich participation, high attendance, and inclusion thanks to the proximity to the workplace and the opportunity to play during work time. This was also acknowledged by participants.

If it had taken place outside working hours then even fewer would have wanted to participate but there actually were quite a few more who wanted to participate (than we had space for)…. (P1)

The sport setting as space affected the mood of the participants, where expressions of improvement and competition where present. Entering the sports room with training gear created a different mood compared to being in working clothes.

It is a big difference to be in the ping pong room with workout clothes and shoes, not the ordinary protective shoes and stuff … everyone talked about it and they got a little excited. It was a pretty good pace and it was fun. Started with warm up; round ping pong. Since I won all the time it was great fun [laughter] … it is exciting and fun, it is a little strenuous and there is a competitive part in it. (P1)

However, the feeling of competition in the room was also experienced as excluding.

For me, it was too hard, I never played. It went too fast. Everyone else was better, so who could I play against? (P3)

The intervention, to a varying extent, affected the workload. Most participants resolved the extra workload with cooperation, where colleagues covered for each other, and with individual re-planning.

We planned our work a little better which gave me the time needed. (P3)

In some cases, participation created increased stress when participants came back to the workplace after the training session.

There are a lot of deadlines during the day that needs to be addressed, which is no 1. I managed to solve it but felt really stressed. I had to catch up when I got back. There must be more margins. I can’t be replaced so quickly. (P2)

The fact that the intervention was conducted in a space at the workplace caught the interest from many the other employees who were not participating. Some of the other employees were jealous and annoyed that they missed the offer to participate, and some showed interest in what was going on.

Some have shown interest and asked what we do, what we learn, what we get out of it. A little sarcastic sometimes, but they know that we have practice and that we are learning how to play ping pong. Some people are curious, they would have liked to be there too … no one goes in there by themselves if the room is empty. But if anyone is in there playing, laughing and having fun it attracts more. (P6)

In summary, the participants were quite homogeneous in terms of readiness for competition mood, but there was an element of omission for those who felt excluded from the competitive mood of the room. Nevertheless, having accessibility and being allowed to participate on working hours was crucial for implementation of the intervention.

## Discussion and conclusion

The aim of this study was to elucidate the participant’s experiences of how health, well-being, and organizational culture were affected by a sport-based health intervention at the workplace. Being inspired by hermeneutic phenomenological analysis, the ambition was not to gain insight into specific aspects of these experiences in order to highlight and problematize the complexity (Vagle, 2009; van Manen, 1997). But rather for themes to emerge from the analysis of participants experiences, where each theme has a distinct direction but at the same time, somewhat overlapping. We have emphasized internal validity, which means making conscious decisions throughout the research process (Kvale, 2007; Miles & Huberman, 1994). This requires, according to Whittemore, Chase, and Mandle (2001) authenticity and integrity, which has been important in this process. This study has limitations; firstly, there is a risk that the intervention selection was biased since it was an open request to all 150 employees, and it turned out that most of the participants had a background in sports. Secondly, nine respondents in the focus interviews was a small number, but based on the premises for selection this was the conditions for the study. Internal validity is a prerequi-site for transferability (Guba & Lincoln, 1981; Polit & Beck, 2010), which means that the accuracy of the results can be transferred to other, but similar, contexts than the one currently studied. Despite the limitations in this study, the findings may be applicable to, and have currency in other similar con-texts, an argument supported by for instance Kvale (2007) and Yin (2014). Three themes emerged in the analysis; *Expressing positive individual effects, Expressing improved work environment* and *The meaning of the workplace as lived space*. The themes will be discussed in relation to the intervention’s three basic health focuses; physical activity, social relations and learning.

The participants expressed positive experiences in terms of feeling enjoyment in learning and competing in table-tennis, feeling increased social connectedness, and improved general well-being which is in line with studies by Perkins and Williamon (2014) and Lamu and Olsen (2016). The intervention, in accordance with Watson et al. (2018), focused on developing personal resources, which was acknowledged by the participants. Furthermore, the participants elucidated that social cohesion over departments and common enjoyment as factors that could improve organizational effectiveness (Watson et al., 2018). From an organizational perspective social and sport-based interventions in the workplace may be a powerful tool to enhance employee’s motivation, collaboration, work efficiency, create a more attractive workplace and improve recruitment of new employees (Rajaratnam et al., 2014). The group was, in general, already physically active outside work, nevertheless doing this intervention during working hours was positive for individual well-being as well as social cohesion and the work environment, which is in accordance with Delisle et al. (2010). Only one participant had a sedentary lifestyle, and this intervention affected his physical wellbeing in a positive way and motivated him to be more physically active in general.

The workplace as a lived space for sport yielded a number of advantages. For the individual, it was easy to participate due to the geographical proximity, convenient to leave work or partake directly before or after their shift and motivating to be a part of the social community that arose. For the organization, the workplace as a lived space for sport may be a way to improve the social work environment, wellbeing, recruitment, and productivity. The intervention generated improvements in the culture of health in the organization through physical activity, more open social relations and on work-related learning, which is in line with previous research (Meacham Webb & Krick, 2017; Taylor et al., 2018).

The workplace as a lived space yielded some disadvantages. One disadvantage was the increased workload caused when a colleague was taking part in the intervention. This created some stress for colleagues who had to take that load and for the participant who had to work harder before or after training to balance the extra workload. Although the participants did not experience this as a problem, we did not have the possibility to ask their colleagues about their experience.

The table tennis table is mobile and therefore the physical space can be modified, but there is a risk that space becomes a monofunctional “space of competition” (Eichberg, 1998, 2016). By placing a table at the workplace traditional spaces of competition can be challenged. The lived space is, however, according to Bengtsson (1998) always situated and associated with social and cultural conventions which affect the quality of the lived space, and according to Shilling (2003) we are included in and excluded from communities through our physicality. Even if reassurances were made that enjoyment and play would be central in the current intervention, the dominating mood of the space was based on competition and improvement. The competitive mood can be challenging for ideas of the health-promoting space, which is in line with findings from a study by Mikaelsson, Rutberg, Lindqvist, and Michaelson (2019).

In conclusion, the intervention failed to reach a central target group, the sedentary population. Instead, in general, it reached employees with sports habitus and a positive attitude towards developing sports skills and competition. The choice of activity includes or excludes and when choosing an activity based on competitive sports values a group of people will feel excluded. In order to enhance individual and organization effects, the workplace as a lived space for sport and health, future research should address specific target groups such as employees with a sedentary lifestyle. Participants should be selected according to health needs and be included in the process of selecting a sports or physical activity, which would be in line with Karanika‐Murray and Weyman (2013) and Mikaelsson et al. (2019). Nevertheless, the analysis shows the workplace as a lived space that encompasses table-tennis activities can convey positive impacts on well-being, social relations and learning.
